# Association between glycemic control and albuminuria among Peruvian adults with diabetes mellitus 2: a cross-sectional analytical study

**DOI:** 10.1590/1516-3180.2021.0448.R2.07022022

**Published:** 2022-07-15

**Authors:** Lucero Del Carmen Collazos-Huamán, Camila Guerreros-Espino, Percy Herrera-Añazco, Vicente Aleixandre Benites-Zapata

**Affiliations:** I Undergraduate Student, Faculty of Medicine, Universidad Peruana de Ciencias Aplicadas (UPC), Lima, Peru.; II Undergraduate Student, Faculty of Medicine, Universidad Peruana de Ciencias Aplicadas (UPC), Lima, Peru.; III MD, MHEd. Researcher, Universidad Privada San Juan Bautista (UPSJB), Lima, Peru; and Assistant Manager, EsSalud, Instituto de Evaluación de Tecnologías en Salud e Investigación, Lima, Peru.; IV MD. Researcher, Unidad para la Generación y Síntesis de Evidencias en Salud, Universidad San Ignacio de Loyola (USIL), Lima, Peru.

**Keywords:** Diabetes mellitus, Albuminuria, Glycated hemoglobin A, Diabetes complications, Peru, Chronic kidney disease, Diabetic nephropathy, Kidney disease

## Abstract

**BACKGROUND:**

Albuminuria is a risk factor for microvascular and macrovascular complications in the diabetic population. However, few studies have correlated poor glycemic control and albuminuria prevalence in Hispanic populations.

**OBJECTIVE:**

To evaluate the association between glycemic control and albuminuria among Peruvian adults with type 2 diabetes mellitus (T2DM).

**DESIGN AND SETTING:**

Cross-sectional analytical study among adults with T2DM in Lima, Peru.

**METHODS:**

We included adults over 18 years old who were in a clinical follow-up program at a private clinic in Lima in 2018. Poor glycemic control was defined as a serum value of glycosylated hemoglobin A1C (HbA1C) ≥ 7%. Albuminuria was defined as albumin values > 30 mg/dl in the first morning urine. We generated generalized linear regression models from the Poisson family with robust variance. We calculated the crude and adjusted prevalence ratios (PRs) with their 95% confidence interval (CI).

**RESULTS:**

We analyzed 907 participants of median age 58 years (interquartile range, IQR 49 to 66), and 62.8% were males. The prevalence of poor glycemic control was 39.8%, and the prevalence of albuminuria was 22.7%. The prevalences of albuminuria in groups with poor glycemic control and adequate glycemic control were 32.7% and 16.1%, respectively. In the adjusted regression analysis, we found a statistically significant association between poor glycemic control and albuminuria (annual percentage rate, aPR = 1.70; 95% CI: 1.28-2.27).

**CONCLUSIONS:**

The prevalence of poor glycemic control and albuminuria was high in our study population. Moreover, Peruvian T2DM adults with poor glycemic control were more likely to have albuminuria.

## INTRODUCTION

Type 2 diabetes mellitus (T2DM) is a worldwide public health problem.^1^ The prevalence of T2DM in 2017 was 451 million cases worldwide, and according to the estimate of the International Diabetes Federation for the year 2045, this figure will rise to 693 million people.^2^ Around the world, almost 50% of T2DM cases have not yet been diagnosed.^2^

In Peru, diabetes treatment and control are poor. Regarding management, a study on rural, rural-to-urban migrant and urban participants showed that the proportions of diabetes awareness, treatment and control were 71.1%, 40.6% and 7.7%, respectively.^3^ In another study on ambulatory T2DM patients at a public hospital in Lima, almost seven out of ten patients had abnormal glycemic control.^4^ At the primary care level, one study found that 20 to 30% of diabetic patients who knew about their disease were not following any type of treatment and had had a late diagnosis, given that 68% of the cases knew that their diagnosis had only been made because of the complications of T2DM.^5^

In this context, complications relating to T2DM among Peruvian adults, such as retinopathy, cardiovascular disease, neuropathy and kidney disease, are an important target for public health strategies.^6^ However, there are structural problems in the Peruvian healthcare system that limit adequate care for diabetes patients.^7^ In addition to the poor quality of clinical practice guidelines for diabetes, there is also a lack of diagnostic methods and medications in primary care centers for managing these patients.^8,9^

Albuminuria, along with a decreased glomerular filtration rate, is a component of diabetic kidney disease and is a risk factor for mortality and cardiac and ocular complications among diabetic people.^10,11,12^ There are several risk factors for albuminuria, such as duration of diabetes, male gender, creatinine levels and poor glycemic control, among other variables.^13^ Glycosylated hemoglobin A1C (HbA1c) is a glycemic control marker. This marker has a positive correlation with blood glucose levels during the previous six to ten weeks. Higher levels are associated with an increased risk of developing microangiopathy among diabetics.^14^

In Peru, the prevalence of albuminuria is high in the population at risk. A study in a screening campaign in 23 nephrology centers nationwide found that the frequency of microalbuminuria was 53.45%, and that 8.96% of the patients had microalbuminuria > 100 mg/dl.^15^ In another study in a primary care center, the prevalence of albuminuria was 17.9% among diabetes patients and 10.8% among hypertension patients.^16^

Poor glycemic control has been correlated with albuminuria in various studies using HbA1C,^17,18,19,20,21^ while intensive glucose control reduces the risk of albuminuria.^21^ However, although studies have been conducted in different ethnic groups, few studies have included any significant proportion of Hispanic patients, despite the evidence that there are ethnic variations in albuminuria prevalence among diabetic patients.^22,23^ Some studies have found that Hispanic patients have greater probability of albuminuria or the initial stages of diabetic kidney disease than other ethnic groups.^24,25^ Considering the burden of diabetic kidney disease in the Hispanic population, and that studies that have included the Hispanic population have been conducted in the United States and not in Latin America, under the conditions of a different healthcare system that could condition different health outcomes,^26^ studies on this disease in this ethnic group are needed.^27^

## OBJECTIVE

Thus, we aimed to evaluate the association between glycemic control and albuminuria among Peruvian adults with T2DM.

## METHODS

### Study design and population

We conducted a study with an analytical cross-sectional design. We included adults over 18 years of age with T2DM who attended a healthcare program called “Take Care” at a private clinic in Lima, Peru, in 2018.

“Take Care” is a healthcare program among chronic patients previously diagnosed with T2DM, arterial hypertension, dyslipidemia or asthma, with comprehensive monthly follow-up controls. The program offered by each patient’s insurance policy covers laboratory tests, procedures and medical consultations, according to that patient’s comorbidities. In addition, the clinical staff register all the patient’s information in the database of the program in order to carry out personalized follow-up and provide adequate treatment.

We excluded patients whose data were incomplete or poorly recorded in the database and patients with a history of arterial hypertension and chronic kidney disease.

### Sampling and calculation of sample size

To calculate the sample size, we used a study in which albuminuria and HbA1C among type 2 diabetic patients was evaluated. This showed that the prevalence of poor glycemic control was 70%.^20^ In addition, in that study, the prevalence of microalbuminuria among participants with poor glycemic control was 57%, while the prevalence of microalbuminuria was 28% among participants with adequate glycemic control. With these values and using a 95% confidence level and statistical power of 80%, we calculated a sample size of 110 patients. However, because we had access to the “Take Care” program database, we decided to analyze all participants who met our eligibility criteria during 2018.

### Main variables

Our exposure variable was glycemic control. A serum value for glycosylated HbA1C ≥ 7% was defined as indicative of poor glycemic control. Our outcome variable was the presence of albuminuria, defined as its presence in the first morning urine, considering values > 30 mg/dl as positive results.^28^ We considered the following as potential confounding variables: age, sex, systolic blood pressure (SBP) and diastolic blood pressure (DBP) (mmHg), fasting glucose (mg/dl), uric acid (mg/dl), creatinine (mg/dl), waist circumference (cm) and body mass index (BMI) (kg/m^2^).

### Data collection procedure

In the “Take care” healthcare program, the anamnesis and physical examination were carried out and recorded in the electronic medical record during the consultation with the physician. Subsequently, the clinical staff grouped the electronic medical records and laboratory tests in the healthcare program database.

We requested the database of the patients with T2DM who had undergone a check-up within the “Take care” healthcare program in 2018. These patients had attended at least one annual check-up; for this purpose, laboratory tests were performed two days before they saw the physician.

We reviewed the database and eliminated patients whose data were incomplete. We considered the HbA1C data that coincided with the date on which the participants underwent the albuminuria test. Moreover, we considered only the first annual measurement of both of these variables. In the same way, other laboratory tests were conducted on the same date on which the participants underwent an albuminuria test. The laboratory method for measuring glycosylated hemoglobin consisted of high-resolution chromatography, and the immunoturbidimetric method was used for albuminuria.

### Ethical considerations

The Institutional Review Board of the Universidad Peruana de Ciencias Aplicadas approved the research protocol on March 31, 2020 (PI 113-18). This study did not have identification codes for the participants, which thus maintained the confidentiality of patient information. The information used in this study was handled solely and exclusively by the authors of this study. Similarly, permission for collection of information from the database of the private clinic was obtained from the ethics committee of the private clinic, which approved the handling of data and its publication (letter no. 006-TI-UDID-CI-2019).

### Statistical analysis

We used the mean and standard deviation to describe the numerical variables with normal distribution. For variables with skewed distribution, we used the median and interquartile range (IQR). We used absolute and relative frequencies for categorical variables.

We used Student’s t test to compare numerical variables with normal distribution, and for numerical variables with skewed distribution, we performed the Mann-Whitney U test. We used the chi-square test to compare categorical variables and correlated numerical variables using the Pearson coefficient.

We generated crude and adjusted generalized linear models from the Poisson family with robust variance to assess the association between poor glycemic control and albuminuria. We reported the prevalence ratio (PR) as an association measurement, with the respective 95% confidence interval (CI). As described in the literature, we entered potential confounding variables into the multivariable model using an epidemiological approach.^29^ Additionally, we evaluated collinearity between the variables before entering them into the multivariable model.

We carried out all analyses in the STATA statistical package (Statacorp, College Station, Texas, United States), version 14.0.

## RESULTS

In total, we analyzed 907 participants; the majority of the sample was male (62.8%). The participants’ median age was 58 years (IQR 49 to 66) and their median BMI was 29.05 kg/m^2^ (IQR 26.6 to 32.3). The median HbA1C was 6.6% (IQR 6 to 7.9) and the median albuminuria was 12.9 mg/dl (IQR 6.02 to 26.1). The prevalence of poor glycemic control was 39.8% (n = 361) (Table 1).

**Table 1.** Clinical and demographic characteristics of the study population according to glycemic control

Variable	Whole sample (n = 907)	Poor glycemic control (n = 361)	Good glycemic control (n = 546)	P-value
Albuminuria (mg/dl)	12.9 (6.02-26.1)	17.2 (7.32-43.6)	10.7 (5.55-20.1)	< 0.01
Age (years)	58 (49-66)	56 (47-64)	58 (49-67)	< 0.01
Gender				0.01
Female	337 (37.2)	116 (32.1)	221 (40.5)	
Male	570 (62.8)	245 (67.9)	325 (59.5)	
Fasting glucose (mg/dl)	122 (106-150)	157 (125-200)	114 (101-126)	< 0.01
BMI (kg/cm^2^)	29.1 (26.6-32.3)	29 (26.8-32.5)	29.1 (26.2-32.1)	0.36
Uric acid (mg/dl)	5.2 (4.2-6.1)	4.7 (3.8-5.6)	5.4 (4.6-6.2)	< 0.01
Creatinine (mg/dl)	0.79 (0.67-0.94)	0.78 (0.66-0.91)	0.8 (0.68-0.95)	0.07
WC (cm)	101 (10.7)	102 (10.2)	100 (11.0)	0.02
SBP (mmHg)	117 (10.5)	119 (9.9)	116 (10.7)	< 0.01
DBP (mmHg)	72 (7.7)	73 (7.9)	71 (7.5)	< 0.01

Values are presented as mean (standard deviation), median (interquartile range) or number (percentage). BMI = body mass index; DBP = diastolic blood pressure; SBP = systolic blood pressure; WC = waist circumference.

**Figure 1.**
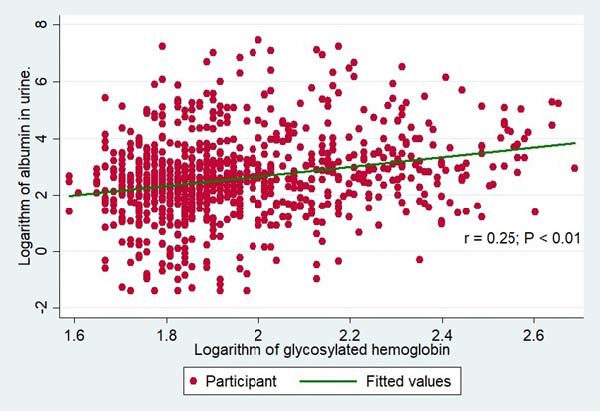
Scatter plot between the logarithms of glycosylated hemoglobin and serum albumin.

The median albuminuria was higher among individuals with poor glycemic control (17.2 mg/dl; IQR 7.32 to 43.6) than among those with adequate glycemic control (10.7 mg/dl; IQR 5.55 to 20.1), and this difference was statistically significant (P < 0.01). The logarithms of the albuminuria and glycosylated hemoglobin levels showed a positive and statistically significant correlation (r = 0.25; P < 0.01) (Figure 1). Also, we found higher medians of fasting glucose, creatinine, waist circumference, SBP and DBP in the group with poor glycemic control than in the group with adequate glycemic control (P < 0.05). The demographic, laboratory and clinical variables referring to the population are described in Table 1.

The prevalence of albuminuria was 22.7% and was higher among men (25.6% versus 17.8%, P < 0.01). In addition, there was higher median fasting glucose, abdominal circumference and SBP in the group with albuminuria than in the group without albuminuria (P < 0.01). The prevalences of albuminuria in the groups with poor glycemic control and adequate glycemic control were 32.7% and 18.1% (P < 0.01), respectively. Table 2 shows the differences in the study population according to albuminuria.

**Table 2.** Clinical and demographic characteristics of the study population according to albuminuria status

Variable	Albuminuria (n = 206)	No albuminuria (n = 701)	P-value
Poor glycemic control	118 (32.7)	243 (67.3)	< 0.01
Adequate glycemic control	88 (16.1)	458 (83.9)	
Age (years)	57 (48-65)	58 (49-66)	0.43
Gender			< 0.01
Female	60 (17.8)	277 (82.2)	
Male	146 (25.6)	424 (74.4)	
Fasting glucose (mg/dl)	133 (109-181)	120 (106-144)	< 0.01
BMI (kg/cm^2^)	29.3 (27.1-32.7)	28.9 (26.3-32.2)	0.09
Uric acid (mg/dl)	5.1 (4.2-6.1)	5.2 (4.3-6.0)	0.72
Creatinine (mg/dl)	0.8 (0.7-1.0)	0.8 (0.7-0.9)	0.11
WC (cm)	103 (9.6)	100 (11.0)	< 0.01
SBP (mmHg)	120 (10.2)	116 (10.5)	< 0.01
DBP (mmHg)	73 (8.1)	71 (7.6)	0.05

Values are presented as mean (standard deviation), median (interquartile range) or number (percentage). BMI = body mass index; DBP = diastolic blood pressure; SBP = systolic blood pressure; WC = waist circumference.

The crude regression analysis showed an association between poor glycemic control and albuminuria (PR = 2.03; 95% CI: 1.59-2.58). After adjusting for age, sex, SBP, DBP, fasting glucose, uric acid, BMI and creatinine in the multivariable analysis, the association with poor glycemic control remained statistically significant (PR = 1.48; 95% CI: 1.19 -1.85) (Table 3).

**Table 3.** Crude and adjusted regression models for the association between glycemic control and albuminuria

Variable	Crude PR (95% CI)	P-value	Adjusted PR (95% CI)	P-value
*Glycemic control*				
Adequate glycemic control	Reference	--	Reference	--
Poor glycemic control	2.03 (1.59-2.58)	< 0.01	1.70 (1.28-2.27)	< 0.01
Age (years)	0.99 (0.98-1.01)	0.52	1.00 (0.99-1.01)	0.89
*Gender*				
Female	Reference	--	Reference	--
Male	1.44 (1.10-1.88)	< 0.01	1.31 (0.99-1.75)	0.06
Fasting glucose (mg/dl)*	1.04 (1.03-1.06)	< 0.01	1.02 (1.01-1.04)	0.02
BMI (kg/cm^2^)	1.02 (0.99-1.04)	0.07	1.01 (0.99-1.04)	0.21
Uric acid (mg/dl)	0.99 (0.91-1.07)	0.83	1.02 (0.95-1.10)	0.60
Creatinine (mg/dl)	1.15 (1.10-1.21)	< 0.01	1.11 (1.04-1.18)	0.01
WC (cm)**	1.02 (1.01-1.03)	< 0.01	–	
SBP (mmHg)	1.01 (1.01-1.03)	< 0.01	1.02 (1.01-1.03)	< 0.01
DBP (mmHg)	1.02 (0.99-1.03)	0.05	0.99 (0.98-1.01)	0.62

*Scaled variable for an increase of 10 mg/dl; **Variable not entered into the adjusted regression model due to collinearity with BMI. BMI = body mass index; DBP = diastolic blood pressure; PR = prevalence ratio; SBP = systolic blood pressure; WC = waist circumference; CI = confidence interval.

## DISCUSSION

The main finding from our study was an association between poor glycemic control and albuminuria in our population. Additionally, almost one third of the sample studied had poor glycemic control or albuminuria.

Two out of every ten patients with DM had albuminuria. In a previous study in Peru, the prevalence of albuminuria was 13.4%, in hospitals in Arequipa.^30^ In a multicenter study on diabetic patients who attended their first nephrological consultation in four hospitals in Lima, 69.3% of them had albuminuria greater than 30 mg/24 hours.^31^ The prevalence of albuminuria in our study was higher than in high-income countries, probably because fewer T2DM patients achieve control over their disease through healthcare services.^32,33^

The prevalence of albuminuria has been found to vary significantly in other countries. Rates of 19.8% to 36.3% were found in southern India and 25.5% in northern India.^34,35,36^ Also, the prevalence of albuminuria was found to be 13.4% in China,^37^ 16.8% in Saudi Arabia^38^ and 24.9% in the United Kingdom.^39^ The differences found are likely to have been due to the sample size, sampling and sample characteristics or, especially, the albuminuria measurement method. For example, while albuminuria was measured with using the first morning urine in our study, the study by Herrera et al., also conducted in Lima, used 24-hour urine. Thus, our findings may have been underestimates.^31^

Our results regarding the association between poor glycemic control and albuminuria were similar to those reported in recent studies conducted in India,^20,40^ Iran,^13^ Nigeria^8^ and Pakistan.^18^ In contrast, although a cross-sectional study conducted in Nepal in 2015 showed a positive correlation between albuminuria and HbA1c, this was not statistically significant; the difference in results was likely to have been due to the limited sample size, as the authors of that study acknowledged.^41^

No specific studies have evaluated this association in the Hispanic population, to the best of our knowledge, although there is evidence suggesting ethnic variation in the prevalence of albuminuria. For example, a study using the National Health and Nutrition Examination Survey (NHANES), on 2,310 diabetic patients, found that the prevalence of early chronic kidney disease (CKD) was greater among Hispanics and African Americans than among whites, and Hispanics had higher albuminuria.^24^ In another study that used NHANES, it was found that among individuals without diabetes, blacks had 2.18-fold and Mexican Americans had 1.81-fold greater odds of having albuminuria than whites, after adjustment for potential confounding factors.^25^ Although the reasons for this variation are not entirely clear, they may be related to the reasons why diabetes and its complications are more frequent in the Hispanic population. These include biological factors, such as predisposition to insulin resistance, augmented insulin secretion and abdominal obesity, as well as complex socioeconomic and cultural factors.^42,43,44,45^

Among patients with poor glycemic control, hemodynamic and metabolic changes to the glomerulus occur, which damage endothelial cells and podocytes and alter the glomerular basement membrane properties, thus contributing to alteration of the physical-chemical characteristics of the glomerular filtration barrier.^46^ In diabetic kidney disease, the glomerular basement membrane loses negative ionic charges and both endothelial and podocyte lesions increase the size of the pores, which thus causes loss of selectivity of glomerular filtration and produces albuminuria.^47^

Clinicians need to emphasize the importance of correct glycemic control among patients with T2DM. The Kidney Disease: Improving Global Outcomes (KDIGO) Diabetes Work Group recommends an individualized HbA1c target ranging from < 6.5% to < 8.0% among patients with diabetic kidney disease that is not being treated with dialysis, in order to reduce the risk of microvascular and macrovascular complications.^48^ Combining interventions such as medication compliance, increased physical activity and healthy nutritional habits can reduce microvascular complications and improve the quality of life of patients with T2DM.^39–41^ Clinicians need to seek periodic measurement of albuminuria levels, especially among patients with poor glycemic control.^28^

Despite this recommendation, glycemic control is not always achieved. In some studies in Peru, it was found that in diabetic patients treated in city hospitals, between 40% and 70% had an HbA1C level greater than 7%.^4,31^ Similar findings have been obtained in other countries such as Russia, China, Myanmar and Angola, where the diabetes control rates were 58.5%, 16.9%, 35.2% and 2.7%, respectively.^49,50,51,52^

A systematic review found two key healthcare system barriers to effective T2DM care and management: financial constraints faced by the patient and limited access to healthcare services and medication. It also found three healthcare system factors that facilitated effective T2DM care and management: use of innovative care models, increased pharmacist involvement in care delivery and education programs led by healthcare professionals.^26^

Our study had some limitations. First, it had a cross-sectional design, which therefore did not allow assessment of causal relationships between the variables. Second, information bias was possible; however, we performed rigorous quality control on the data collected. Third, we did not have any information on some other potential confounding variables, such as the number of years for which the patients had been suffering from T2DM, their physical activity levels, their dietary habits, the length of time since their inclusion in the program and whether they were previously seen at another institution. Similarly, we did not have information on patients’ adherence to the program, or whether they were visiting other doctors outside the program or were hospitalized during the follow-up. Fourth, we unable to use the gold standard for measuring albuminuria (24-hour urine test), which could have caused misclassification of outcomes. However, initial screening of albuminuria using a urine sample collected early in the morning, as was done in our study, has good sensitivity compared with the 24-hour urine test.^53,54^ Lastly, we could not be sure that all the conditions for adequate albuminuria measurement were observed, which might have produced false positives in our sample.

## CONCLUSION

There was an association between poor glycemic control and higher prevalence of albuminuria among Peruvian patients with T2DM. Therefore, we recommend further research on cost-effective glycemic control interventions, in order to reduce the risk of microvascular and macrovascular complications in the Hispanic population.
